# Prevalence of Undiagnosed Attention Deficit Hyperactivity Disorder (ADHD) Symptoms in the Young Adult Population of the United Arab Emirates: A National Cross-Sectional Study

**DOI:** 10.1007/s44197-023-00167-4

**Published:** 2023-12-11

**Authors:** Nabeel Al-Yateem, Shameran Slewa-Younan, Aram Halimi, Sara Aideed Saeed, Daniah Tliti, Muna Mohammad, Mada Ridwan, Razan Zeidan, Muna Hasan Hammash, Fatma Refaat Ahmed, Jacqueline Maria Dias, Syed Azizur Rahman, Muhammad Arsyad Subu, Heba Hijazi, Fatemeh Yeganeh, Aaliyah Momani, Mitra Zandi, Richard Mottershead

**Affiliations:** 1 Department of Nursing, College of Health Sciences, University of Sharjah, P.O.B 27272, Sharjah, United Arab Emirates; 2 Faculty of Science and Health, School of Nursing, Paramedicine and Healthcare Sciences, Charles Sturt University, Orange, NSW Australia; 3 School of Medicine, Western Sydney University, Sydney, Australia; 4 Department of Epidemiology, School of Public Health and Safety, Shahid Beheshti University of Medical Sciences, Tehran, Iran; 5 Research Center for Social Determinants of Health, Research Institute for Endocrine Sciences, Shahid Beheshti University of Medical Sciences, Tehran, Iran; 6 Critical Care and Emergency Nursing Department, Faculty of Nursing, Alexandria University, Alexandria, Egypt; 7 Department of Health Care Management, College of Health Sciences, University of Sharjah, Sharjah, United Arab Emirates; 8 Department of Health Management and Policy, Faculty of Medicine, Jordan University of Science and Technology, Irbid, Jordan; 9 Daphne Cockwell School of Nursing, G, Raymond Chang School of Continuing Education, Toronto Metropolitan University, Toronto, Canada; 10 Maternal and Children Nursing Health Department, Faculty of Nursing, Applied Science Private University, 21 Al Arab St, Amman, Jordan; 11 Department of Medical Surgical Nursing, School of Nursing and Midwifery, Shahid Beheshti University of Medical Sciences, Tehran, Iran

**Keywords:** Attention deficit hyperactivity disorder, Prevalence, Young adult, Academic performance, United Arab Emirates, Mental health stigma

## Abstract

**Background:**

Attention deficit hyperactivity disorder (ADHD), a globally prevalent behavioural disorder, remains underdiagnosed, particularly among adults. This issue is exacerbated in the Arab region due to stigma and insufficient healthcare facilities and professionals. Despite the United Arab Emirates (UAE) efforts to improve mental healthcare, shortcomings persist. No studies in the UAE currently assesses the appropriateness of the screening system for ADHD and other behavioural issues. Furthermore, prevalence rates of ADHD, particularly within the young adult population, are absent.

**Aim:**

To estimate the prevalence of ADHD amongst young adults attending university in UAE and examine its relationship with gender and academic outcomes.

**Methods:**

A cross-sectional, correlational design was used. Young adults in their first year at university were recruited from different academic institutions across the UAE. The study utilized the Adult ADHD Self-Report Scale (ASRS) for data collection.

**Results:**

A sample of 406 young adults, aged between 18 and 20 years of age were recruited. Approximately, 34.7% (*n* = 141) reported symptoms suggestive of probable ADHD. Significantly lower grade point average marks were observed in participants with ADHD symptoms (*M* = 3.15) compared to those without (*M* = 3.35). Females reported symptoms of probable ADHD at higher rates than males, indicating possibly a potential screening deficiency and a potential stigma consequence.

**Conclusions:**

The study demonstrates a high prevalence of probable ADHD in young adults, particularly among females attending university in the United Arab Emirates. Implications for early screening, service provision, and greater professional health training on this disorder are required.

## Introduction and Background

Attention deficit hyperactivity disorder (ADHD) is recognized as the most common behavioural problem in children internationally, including in the UAE and the Arab region. While global estimates suggest a prevalence ranging between 2 and 7% [1, 2], there are no recent studies that have measured the prevalence of ADHD among the UAE population. The most recent studies specific to the UAE were conducted in 2009 by Eapen and in 2011 by Khamais [3, 4], indicating prevalence rates ranging from 4.1% to 12.5%, respectively. In the neighbouring country of Saudi Arabia, a recent meta-analysis by Aljadani in 2023 reported a pooled prevalence of ADHD at 12.4% [5]. Research has demonstrated that screening for, diagnosis, and documentation of ADHD can vary and is influenced by multiple factors such as stigma and healthcare professionals' training. This variability can lead to ADHD being underrecognized and underdiagnosed in many countries, including those in the Arab region [1, 2].

ADHD often persists into adulthood, potentially remaining undiagnosed in this population, leading to a high prevalence of undiagnosed ADHD among adults [6]. A global systematic review and meta-analysis found that the prevalence of persistent adult ADHD (with a childhood onset) was 2.58%, and that of symptomatic adult ADHD (regardless of childhood onset) was 6.76%. This translates to approximately 139.84 million and 366.33 million affected adults globally in 2020 [7]. In a recent systematic review and meta-analysis which examined outpatient psychiatric clinics populations, even higher rates of adult ADHD were noted, ranging from 14.61 to 26.7% depending on the diagnosis method. The study concluded that there are high rates of adult ADHD among psychiatric outpatient clinics, confirming again that many adults with ADHD may be undiagnosed or underdiagnosed [8].

ADHD is characterized by persistent patterns of inattention, hyperactivity, and impulsivity that interfere with functioning or development [9]. In adolescents and young adults, ADHD can profoundly impact various aspects of life and increase the risk for other mental health disorders, leading to negative outcomes such as educational underachievement, difficulties with employment and relationships, and criminality [1, 10]. It is also worth noting that ADHD often co-occurs with other conditions, including oppositional behaviour, anxiety, and social and emotional problems, which can further complicate the prognosis and management of this disorder [11].

The lack of diagnosis, combined with the profound functional impact of ADHD in adults, underscores the need for increased awareness, improved diagnostic procedures, and effective treatment strategies for these conditions. Research has shown that mental disorders are a leading cause of disability in the Arab region. However, Arab countries face a shortage of trained healthcare professionals and treatment facilities, along with a dearth of reliable and valid assessment tools for early screening and assessment, and the lack of the integration of mental health services into the primary healthcare system further compound mental health care in the Arab world [12, 13].

Mental health and behavioural problems are often stigmatized in many cultures around the world, with the Arab region and the Middle East being a notable example. A systematic review of the literature on stigma associated with mental illness in Arab culture found a large diversity in stigmatizing beliefs, actions, and attitudes towards the treatment of mental illness within the Arab population [14]. Limited knowledge and capacity among primary healthcare professionals to identify and diagnose mental health and behavioural problems has also been reported [15, 16].

Over the last few years, there has been concentrated efforts on the part of the UAE government to improve the healthcare system, including mental health care services., such as integrating mental health into primary healthcare settings and a focus on providing specialist mental health services [17, 18]. The UAE has also established school nursing clinics with the aim to promote the health of school-aged children through early screening and prevention of illness [19]. Nonetheless, there is evidence to suggest that further efforts are required including strengthen the care services provided in these school clinics, and addressing the persistent shortage of mental health professionals and enduring cultural attitudes towards mental health that can impacting help-seeking behaviour. Moreover, given the rates of behavioural problems in children and adolescents, specialized services that are capable of screening and identifying potential diagnoses are required [20–22]. This is because early screening for ADHD and other mental and behavioural problems in children is of utmost importance, as it can significantly improve the outcome of treatment and the long-term prognosis of these conditions into adulthood [23]. Once identified, psychosocial interventions are recommended as the first-line treatment for preschool children (4–5 years old) and can be used as adjunct therapy in elementary school children (6–11 years old) and adolescents (12–17 years old) [24, 25]. These interventions can include behaviour therapy, parent training, and classroom accommodations. Early intervention has been shown to improve overall social and cognitive function more broadly and specifically can lead to better academic performance, improved self-esteem, and increased overall well-being. What is more, there is research to indicate that by deploying suitable treatment and support during childhood, the trajectory of ADHD can be altered, potentially reducing the impact of these negative outcomes in adulthood [1].

Given the importance of early screening and diagnosis of ADHD in childhood and the potential limitations in the UAE mental healthcare services, this study sought to examine early screening and diagnosis efforts for ADHD by evaluating the rates of undiagnosed ADHD-like symptoms in the young adult population in the UAE.

### Study aim

The aim of the study was to estimate the prevalence rates of undiagnosed ADHD-like symptoms among young adults residing in the UAE, irrespective of their nationality. Further, the study also sought to examine the impact of these rates on clinical correlates, such as gender differences and academic achievement of the participants.

## Methods

This study used a quantitative, cross-sectional, correlational design. This design is the most appropriate to estimate prevalence.

### Sample and Setting

The study included young adults who had graduated from the school system in the UAE and commenced their university studies, with most in their first year of their studies. These students had moved beyond the health screening system usually implemented in schools and primary healthcare centres and were now adults. To examine rates of undiagnosed ADHD symptoms, participants who self-reported a formal diagnosis of ADHD by a healthcare professional were excluded from the study. This was ascertained through a screening question at the beginning of our survey. In determining our sample size, we factored in a 5% margin of error, a 95% confidence level, and an estimated population size of 3 million for children and young adults below the age of 24 years [26]. The required sample size was a minimum of 385 participants. The achieved sample size in this study exceeds this required sample size.

### Data Collection

Following ethical approval to undertake the study, the research team sought approval from four main universities located in the north, middle, and south of the UAE. These universities were approached because they cover the geographical and social diversities present in the UAE, and their student body represents all backgrounds found in the country. All four universities granted permission to collect data.

Students were invited to participate in the study and were presented with a QR code directing them to the online survey. The first page of the survey consisted of a consent form, and they could only proceed with the survey if they agreed to the ‘participate’ option of the consent form. The research team remained nearby as the participants completed the forms, ready to assist if they faced any difficulties, answer their research-related questions, and encourage them to answer all the questions. Data collection took place in the period between February and April 2023.

### Measure

The Adult ADHD Self-Report Scale (ASRS) is a commonly used scale to screen for attention deficit hyperactivity disorder in adults. It was developed by the World Health Organization (WHO) in collaboration with the Workgroup on Adult ADHD and consists of 18 items that correspond to the 18 DSM-IV criteria for ADHD [27]. Participants are asked to answer the questions based on how they have felt and conducted themselves over the past 6 months. Participants rate themselves on a five-point Likert scale with options ranging from "never," "rarely," "sometimes," "often," to "very often." For readers' clarity, here are a few sample questions from the ASRS:

- How often do you have trouble wrapping up the final details of a project, once the challenging parts have been done?
- How often do you have difficulty getting things in order when you have to do a task that requires organization?
- How often do you have problems remembering appointments or obligations?

**Fig. 1:**
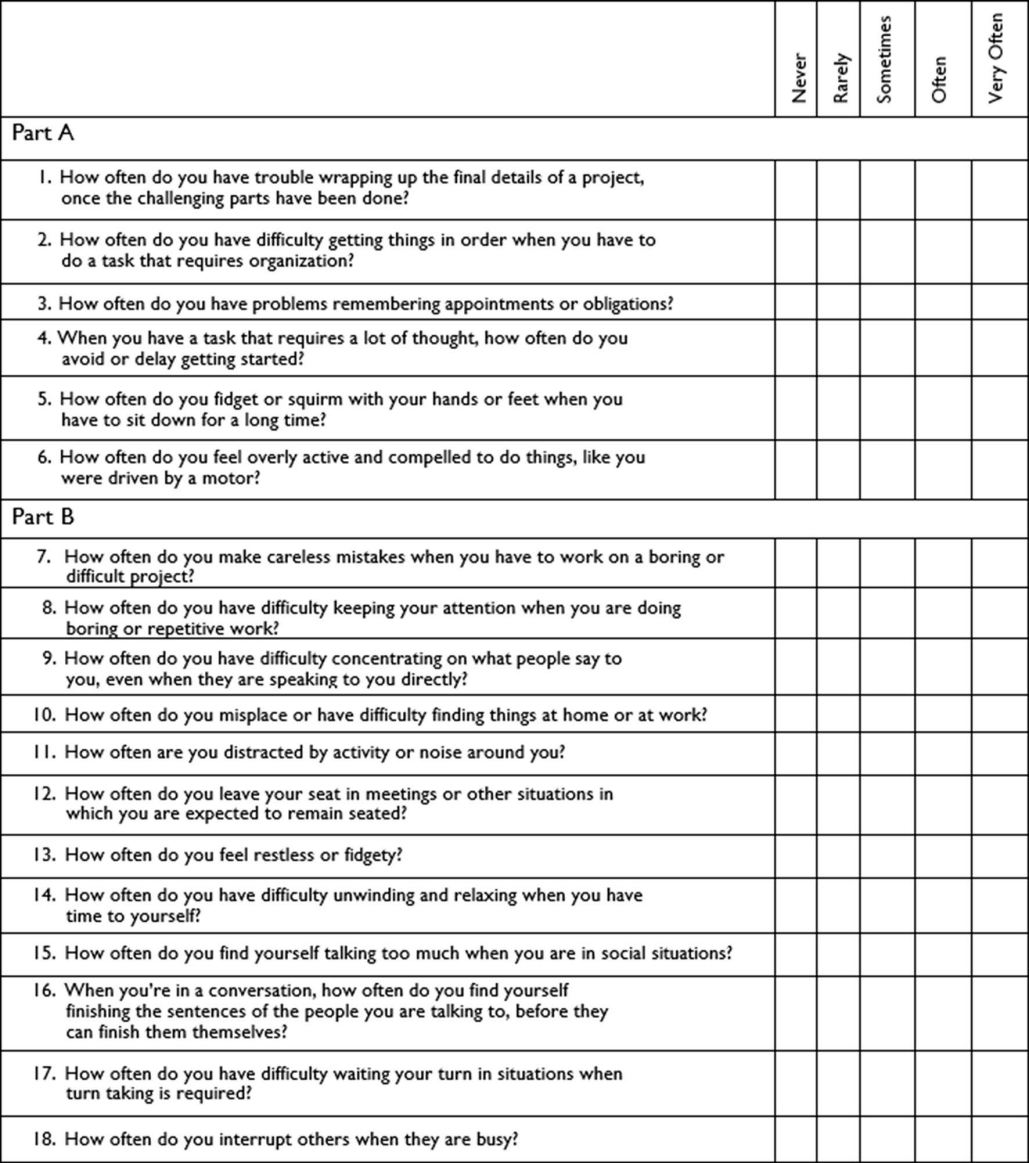
ASRS Checklist

For a comprehensive view of the ASRS checklist, please refer to Fig. 1.

Psychometric properties include the concurrent validity of the ASRS, which when compared to diagnostic interviews for ADHD had a sensitivity score of 68.7% and a specificity score of 99.5% (Kessler et al., 2005). The discriminant validity of the scale has also been established, showing that the ASRS can effectively differentiate ADHD from conditions such as depression and anxiety [28].

The Cronbach's alpha coefficients of the scale were measured in different studies and ranged between 0.63 and 0.72 [27, 29, 30]. The scale has been translated and used with an Arabic-speaking population, and the Cronbach's alpha for the Arabic version of the scale was measured at 0.717 [2, 30–32].

The reliability and validity of both the Arabic and English versions of the scale were initially checked in this study before the full-scale investigation was conducted. Once the reliability and validity were established, the full study was carried out. After data collection was completed and during the initial stages of data analysis, the Cronbach's alpha was recalculated to confirm reliability. The Cronbach's Alpha for the scale was 0.78 at the beginning of data collection, and it increased to 0.861 at the conclusion of the data collection and the commencement of the final analysis. In addition, both item-to-item and item-to-scale correlations were assessed. All items on the scale had a positive moderate correlation with each other.

### Ethical Consideration

Ethical approval was obtained from the University of Sharjah Research Ethics Committee (Ref: REC-23-03-04-03-S). The questionnaire was electronic, and the first page contained the consent to participate form. Participants could not proceed with the study unless they agreed and selected the "agree to participate" option. The study was conducted fully anonymously; no personal data was collected from participants.

Finally, study data was kept confidential, and access was restricted solely to the study team. The data was stored on password-protected computers and drives.

### Data Analysis

First, the Cronbach alpha coefficient was calculated. The item-to-item correlation and the item-to-total scale correlations were calculated to assess the internal consistency and construct validity of the scale.

Descriptive statistics were then constructed to describe the study sample, their demographics, and the prevalence of ADHD symptoms among the sample and the subgroups. The prevalence of ADHD consistent symptoms among participants was calculated using two methods reported in the literature. The first method is based on the initial six items in the scale considered to be a strong predictor of the ADHD symptoms among participants. Within this method, if participant shows more than four positive symptoms this indicates person with probable ADHD. The second method of calculation is to consider all the items of the scale. Utilizing this method, a participant is considered to have ADHD-like symptoms if they had positive responses either to six or more inattention questions or to six or more hyperactivity–impulsivity questions or both [27].

Finally, inferential statistics were performed to assess if there were differences between the participants who had ADHD-like symptoms and others, particularly in relation to academic performance, and to determine if a particular gender had a more prevalent undiagnosed ADHD symptoms than the other group.

## Results

A total of 406 participants were recruited. The majority of the participants (*n* = 286) were aged between 18 and 20 years of age (70%), while the remaining were either 21–24 years of age (20%, *n* = 79) or did not provide answer. Female participants were the majority in the study, comprising 72% (*n* = 294) and the mean academic score for participants reflected by the mean grade point average was 3.28 (SD = 0.5). Table 1 present the study participants’ demographics.

**Table 1:** Participants’ demographic characteristics

Demographic variables	*N*, (%)
Age	
18–20	286 (70%)
21–24	79 (20%)
Gender	
No answer	41 (10%)
Female	294 (72%)
Male	102 (25%)
College	
Did not respond	10 (3%)
Medical and health sciences colleges	202 (50%)
Engineering	86 (21%)
Business, law and humanities	48 (12%)
Did not respond	70 (17%)
Grade point	
Mean = 3.28 (SD = 0.5)	

Within the sample, 34.7% (*n* = 141) reported having more than six symptoms of inattention or hyperactivity or both, indicating probable ADHD. When examining the impact of probable ADHD on academic performance, the GPA scores for participants with ADHD symptoms were lower (*M* = 3.15) compared to those without the symptoms of ADHD (*M* = 3.35), which was statistically significant (*P* < 0.001). When examining differences amongst males and females with probable ADHD, a significantly higher proportion of participants who reported symptoms were females. Table 2 presents distribution of gender across probable ADHD participants and their academic performance (i.e. GPA).

**Table 2:** Distribution of gender across probable ADHD participants and their academic performance (i.e. GPA)

	ADHD negative			ADHD positive			*P*
	Count	Row %	Column %	Count	Row N%	Column %	
Gender							
Female	181	61.6%	70.7%	113	38.4%	80.7%	0.029
Male	75	73.5%	29.3%	27	26.5%	19.3%	
GPA	*M* = 3.35				*M* = 3.15		< 0.001

*Note: This table represents data from 396 participants; 10 participants from the total sample of 406 did not disclose their gender and are not included in the above distribution

## Discussion

The findings of the current study suggest that there is a relatively high rate of probable ADHD amongst young adults in the United Arab Emirates (UAE). Notably, approximately 35.6% of the sample reported having six or more symptoms of inattention or hyperactivity, indicative of potential ADHD. This aligns with previous literature that has suggested ADHD is a significant issue in the region, though it is often underdiagnosed or undiagnosed [1, 8].

A salient finding is that the existing screening services appear to have failed to capture 35% of students who exhibit probable ADHD symptoms, which may be as a result of several factors. Firstly, this may reflect problematic screening processes and thus immediately suggests the need for improved training for healthcare professionals, particularly in primary care settings where many young adults are likely to seek help. Secondly, the stigma associated with ADHD and other mental health disorders may lead to the underreporting of symptoms by the parents of children as they were young, which has been reported as a significant issue in Arab cultures [12, 13].

Our study revealed a notable gender difference, with a larger proportion of female participants identified with ADHD symptoms. While ADHD symptoms might generally be more prevalent in males within the broader population, our findings potentially underscore an underdiagnosed ADHD scenario among females. This underdiagnosis can arise from sociocultural expectations and prevailing attitudes towards female behaviour. The cultural beliefs associated with family honour and marital prospects might lead to reduced reporting of ADHD symptoms by parents of females. Indeed, research suggests that ADHD in girls and women often goes unrecognized, not only due to different symptom manifestations, but also because of societal biases and cultural nuances specific to the region [1].

The observed stigma that may contribute to the underdiagnosis of ADHD could have several sources [33, 34]. Cultural beliefs and attitudes towards mental health issues are likely to play a significant role, with mental health often being a stigmatized topic in the Arab region [35, 36]. It is also important to consider the effect of these cultural attitudes on the family and social networks of individuals. They may be less likely to seek help due to fear of social judgement, which can exacerbate the issue of underdiagnosis. It is also important to note that our study found a significant association between the presence of ADHD symptoms and lower academic performance, measured by GPA. This aligns with prior research suggesting that ADHD can have a substantial impact on academic performance due to difficulties with concentration, organization, and time management [10]. The academic implications of ADHD underline the importance of early diagnosis and intervention, which can help mitigate these negative effects and improve overall outcomes for students.

This study presents valuable insights into the prevalence and impact of ADHD symptoms, particularly in school settings, and the notable percentage of females who may be underdiagnosed due to cultural stigma. It suggests that healthcare professionals should bolster their understanding of these conditions through professional development, with an increased emphasis on the potential variance in ADHD manifestations among different demographics. Schools and higher educational institutions may consider routine screenings for ADHD in their health protocols, as early detection and intervention could result in improved academic and social outcomes.

Moreover, the study suggests the importance of public awareness campaigns to reduce stigma associated with ADHD and increase understanding of the condition. Policymakers could consider directing more resources towards mental health services, enhancing ADHD screening tools, and increasing the availability of professionals trained in this field. Inclusion of ADHD education in healthcare professional curricula is also highlighted, emphasizing its impact on academic performance and the risk of underdiagnosis. Although these findings present a noteworthy direction, further exploratory studies could provide a comprehensive starting point for deeper understanding and more tailored interventions.

## Conclusion

In conclusion, the study demonstrated high rates of probable ADHD among young adults attending university in the United Arab Emirates, particularly among females. These rates may imply limitations in the screening process and suggest the necessity for enhanced professional training, particularly in primary care settings. It is also crucial to address the stigma surrounding ADHD and mental health disorders which may influence symptom reporting and deter help-seeking behaviour, an issue known to be prominent in Arab cultures.

### Limitation of the Study

This study provides an overall estimate of the prevalence of undiagnosed ADHD in the young adult population, but it has limitations, notably concerning selection and information bias. The study was not designed as a typical prevalence study and did not rigorously address these biases, which could impact the applicability and accuracy of the findings.

Selection bias in this study could have arisen from the voluntary participation method used for recruitment. Participants who chose to participate might differ from those who did not, such as already self-identifying as having ADHD symptoms. This self-selection process could potentially affect the results, as it might not represent the broader young adult population accurately. Additionally, the study recruited participants from universities. This introduces a risk of underrepresentation of individuals with more severe ADHD symptoms who might not be attending university due to their condition.

Furthermore, information bias might have influenced the results. Since the study relied on self-reported data, there is a risk of inaccurate or incomplete information. For instance, participants may misreport or underreport their symptoms due to stigma or lack of awareness about ADHD, which could affect the estimate of undiagnosed ADHD prevalence. In conclusion, while the study provides a valuable estimate, further research with a more diverse sample and robust bias management strategies would offer a more accurate and generalizable understanding of undiagnosed ADHD prevalence among young adults.

Finally, another limitation of our study is not directly accounting for the unique challenges faced by first-year students transitioning from school to higher education. The stressors associated with this significant change, from new academic demands to social adjustments, could influence the presence or perception of ADHD-like symptoms. Future studies could benefit from considering such educational transitions when evaluating the prevalence and impact of ADHD symptoms.

## Data Availability

The datasets used and/or analysed during the current study are available from the corresponding author on reasonable request.

## Abbreviations

ADHD: Attention deficit hyperactivity disorder
UAE: United Arab Emirates
ASRS: Adult ADHD Self-Report Scale
WHO: World Health Organization
